# Coup De Soleil, or Stroke of the Sun

**Published:** 1841-10

**Authors:** 


### COUP DE SOLEIL, OR STROKE OF THE SUN.

We observe by the newspapers that Dr. Dowler of New Orleans, formerly of the state of Ohio, has dissected the bodies of two or three persons, who had died from exposure to the sun, and found the lungs, not the brain apoplectic. We write this paragraph, for the purpose of calling attention to cases of this kind. Their pathology has not been properly investigated, and we hope that such of our readers, as may have the opportunity, will not fail to note the symptoms and morbid appearances after death. D.

